# Understanding the Percolation Characteristics of Nonlinear Composite Dielectrics

**DOI:** 10.1038/srep30597

**Published:** 2016-08-01

**Authors:** Xiao Yang, Jun Hu, Shuiming Chen, Jinliang He

**Affiliations:** 1 State Key Lab of Power Systems, Department of Electrical Engineering, Tsinghua University, Beijing, 100084, China

## Abstract

Nonlinear composite dielectrics can function as smart materials for stress control and field grading in all fields of electrical insulations. The percolation process is a significant issue of composite dielectrics. However, the classic percolation theory mainly deals with traditional composites in which the electrical parameters of both insulation matrix and conducting fillers are independent of the applied electric field. This paper measured the nonlinear V-I characteristics of ZnO microvaristors/silicone rubber composites with several filler concentrations around an estimated percolation threshold. For the comparison with the experiment, a new microstructural model is proposed to simulate the nonlinear conducting behavior of the composite dielectrics modified by metal oxide fillers, which is based on the Voronoi network and considers the breakdown feature of the insulation matrix for near percolated composites. Through both experiment and simulation, the interior conducting mechanism and percolation process of the nonlinear composites were presented and a specific percolation threshold was determined as 33%. This work has provided a solution to better understand the characteristics of nonlinear composite dielectrics.

Electrical ageing of polymer dielectrics, which is caused by the effect of high electrical field, is a vital threat to all electronic and electrical elements, devices, and systems. Many effects have been performed to modulate the performance of polymer dielectrics for improving the lifetime and stability of electrical and electronic devices, such as batteries^1^, capacitors^2^,^3^, and microelectronics^4^. Composite insulation with nonlinear electrical parameters can function as smart materials for stress control and field grading in all fields of electronic and electrical insulations, such as in power electronic applications like IGBT modules^5^, and also in electrical systems for polymer insulators^6^, cable accessories^7^, stator coils of electrical machines^8^. Typical nonlinear composites are ZnO microvaristors filled polymers like silicone rubber^9^, epoxy^10^, Polyethylene^11^,^12^, whose nonlinear conducting behavior as well as application approaches have been studied by a number of researches^13^,^14^,^15^.

For better understanding and controlling the field-dependent property of the composites, the percolation process is a significant issue as it indicates how the nonlinear behaviors of composites vary with filler concentration. The percolation threshold is also necessary to be determined so as to get a critical concentration above which the composite begins to exhibit nonlinear properties. Traditional theory concerning the percolation process of the conducting composites mainly deals with the well-known law described as Equation (1) for near percolated cases^16^,^17^.


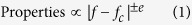


where *f* is the volume fraction of the fillers; *f*_c_ is the percolation threshold; the index *e* is a critical exponent which is different for various properties. For instance, the overall conductivity σ_c_ of a near percolated composite varies with the filler concentration *f* as Equation (2) shows^18^,^19^.


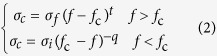


where σ_*f*_ and σ_*i*_ are the conductivities of the fillers and the insulation matrix, respectively. The common approach^20^,^21^ is to measure the conductivity of the composites with different filler concentrations, then by fitting the σ_c_(*f*) curve to Equation (2), the percolation threshold *f*_c_ and critical exponent *t* and *q* are determined. However, this method is not suitable for composites with nonlinear conducting behavior like ZnO microvaristor/Polymer composites. Firstly, Equation (2) fits better to the case that the difference of the conductivity between the fillers and the insulation matrix is large^16^. But when the applied electric field is relative low, the fillers would not exhibit nonlinear property and their conductivities are close to the insulation matrix. Moreover, as we mainly focus on the nonlinear property of the composites, Equation (2) is not necessary to be applied in this case. Then when the field is relative high, some fillers begin to present nonlinear property and the electric field distribution in the composite is complicated, which may be different from place to place. As the conductivities of the fillers depend on the local field, they are in great discrepancy and cannot be used to calculate the overall conductivity of the composite. As the tradition approach of formula fitting is no longer applicable, it’s more appropriate to analyze the nonlinear composites with various filler concentrations near the percolation threshold case by case.

Nevertheless, the percolation threshold of the nonlinear composite has been inconclusive yet. Though the general accepted percolation threshold of composites with spherical fillers is 16%vol^17^, many researches for nonlinear composites have reported much higher values than that. For ZnO/high-density polyethylene^11^, the composite failed to exhibit nonlinear behavior until reaching the filler fraction of 35%vol. EPDM composite filled with rounded SiC filler was reported to present similar percolation threshold of 33%vol. Also some studies concluded that for nonlinear composites with spherical fillers like ZnO and SiC, high filler concentrations of typically 30 ~ 40%vol were required to obtain nonlinear electrical conductivity^22^,^23^, which are far beyond the general accepted value.

To shed light on the interior conducting mechanism of the nonlinear composites, simulation could be conducted for the comparison with the experiment result. Existing models to simulate the conducting behavior as well as percolation process of the nonlinear composites are based on the random checkerboards model^24^, in which fillers are represented by single cubes in a cubic lattice as the insulation matrix. This model can effectively deal with composites containing irregular-shaped fillers but it may be not applicable for those with rounded fillers. This is because the percolation process is influenced by the type of filler-filler contact interfaces, which mainly depend on the particle shapes. For composites with irregular shaped fillers, two types of contacts between fillers must be distinguished, namely face contact and edge contact (point contact can be ignored)^25^. And it is proved by both simulation and experiment^26^ that two types of filler-contacts lead to two percolation thresholds. But for composites with rounded fillers, only one type of contact exists between the spherical particles, shown as ZnO microspherical varistors in Fig. 1. In this case a single percolation threshold is expected. As the fillers in the checkerboards model are represented by squares, a new model considering rounded fillers should be established.

This paper put forward a model based on Voronoi network to simulate the nonlinear V-I characteristics of silicone rubber composites with rounded ZnO microvaristor fillers. The electrical property of the filler, the insulation matrix and the contact interface are all involved in the model. The experimentally measured V-I curves of the composites with different filler concentrations are also acquired to verify the model. Through both experimental and simulation analysis, the percolation process is studied and the percolation threshold of ZnO microvaristor/silicone rubber composites is determined.

## Result and Discussion

### Main Measurement Result

The nonlinear V-I characteristics of ZnO microvaristor /silicone rubber composite samples with different filler concentrations measured under different areas of electrodes are shown in Fig. 2, the simulation results mainly agree with the experimental curves measured under the 20 mm electrode. We define the switching field *E*_b_ as the applied electric field when the current density *J* reaches 10^−5^ A/cm^2^, above which *J* increases steeply to the maximum limit.

For the samples with filler volume concentrations of 39%, 43% and 47%, *E*_b_ determined by experiment and simulation are almost the same and they decrease with the increase of the filler concentration. Samples with above three filler concentrations present typical nonlinear behavior and can be attributed to the fully percolated case. For the 35% sample, the switching field is even higher and it exhibits breakdown feature. This is a sign of approaching percolation and is emphatically discussed in the paper. Finally the 30%, 25% and 20% samples fail to exhibit nonlinear conducting characteristics even under the maximum output of the voltage source, thus they are not presented in Fig. 2.

Above measurement results can be explained through Fig. 3, in which each subfigure corresponds to the curve in Fig. 2 of the same filler concentration. Figure 3(a) presents the SEM images of the composites while Fig. 3(b) exhibits the simulated filler and current density distribution when total current density reaches 30 μA/cm. In SEM images, it is evident that many fillers are aligning in series. The conduction may be obscure since some fillers in the paths are wrapped by silicone rubber. But from the appearance of the silicone rubber matrix we can speculate that there may be fillers covered by it. Though it is impossible to locate exact conduction paths in the SEM images, we can find paths completely or partly formed by the fillers throughout the composites. In Fig. 3(a) the aligned fillers are marked by red lines and silicone rubber layers separating two adjacent fillers are marked by yellow lines. We may consider these marked paths as possible conduction paths and compare them to the simulation results. In Fig. 3(b), darker colors of the polygons represent greater current flowing through the grain. Only polygons in the conduction path are black and others are all in light grey because the current density in the path is much higher than other place. In Fig. 3(a,b) the form of the conduction paths are a little different, but their variation tendency against the filler concentration is similar. This similarity explains why the experimental and the calculated V-I curves fit well. Then the conduction mechanism and the percolation process are analyzed through both the experiment and simulation results.

### Fully percolated case

In the 39%, 43% and 47% samples, fillers form thoroughly connecting paths and the current selects the shortest one to flow through. It is evident that in composites with higher filler concentrations, a short conduction path is more likely to form, which means a lower switching field. Thus both in Fig. 3(a,b) the conduction paths become more and more straightforward from the 39% to 47% samples. As a result, the switching field decreases with the filler concentration.

Also in Fig. 2 we can see that for the same composite sample, measured *J*(*E*) curves under different areas of electrodes are different and the switching field of the curves decreases with the electrode area. This mainly results from the contingency of the conduction paths due to random distribution of the fillers. For the same composite sample under the same applied electric field, the amount of conduction paths will increase when the area of electrode increases. According to the definition of the current density *J* (*J* = *I/S*), *J* should remain the same under the same electric field regardless of the areas of the electrodes. Actually, when the area of electrode increases, not only the amount of conduction paths increases, but also the conduction paths become more straightforward because the current can automatically find the shortest paths from a broader range. This is the reason that the switching field of the curves decreases with the electrode area. For composites with high filler concentrations like the 47%vol sample, the conduction paths are plenty enough for the current to select even under the smallest 5 mm electrode. In this case the contingency is minor and the discrepancy of the *J*(*E*) curves due to different areas of electrodes is relative small. For composites with lower filler concentrations, there are not so many conduction paths and the contingency is greater. Thus when the area of electrode increases the current can find shorter conduction paths and the switching field decreases apparently as the case of 43%vol sample shows. It’s interesting that for the 39%vol sample, a nonlinear *J*(*E*) behavior is not observed under the 5 mm and 10 mm electrode (thus are not presented in Fig. 2) but the composite exhibits standard nonlinear characteristics under bigger electrodes of 15 mm and 20 mm. The reason is that in the 39%vol sample, the conduction path is rather rare and only distributed in the area of 10–20 mm away from the center of the composite sample. This is an extreme case of the contingency of the conduction path.

Furthermore, Fig. 2 shows that *J*(*E*) curves measured under 15 mm and 20 mm are very close for all fully percolated cases. This indicates that when electrode diameter is above 15 mm, the contingency of conduction paths is quite small. Thus the curves measured under the 20 mm electrode can be considered as the experiment result with the least contingency and are most representative for the composite sample with a specific concentration. Also in the simulation, the paper has tried to rule out the contingency of conduction path due to random distribution of the fillers through repeated calculation (elaborated in the Method part). Figure 2 shows that the calculated *J*(*E*) curves of the composites are closest to measured curves under the 20 mm electrode. This indicates that the simulation and the experiment result agree well when the contingency since the random filler distribution is basically ruled out.

### Nearly percolated case

In the 35% sample, both the experimental and the simulated curves show higher switching fields than the 39% sample. But when the applied electric field exceeds a certain value, the current density suddenly rockets to the upper limit, which is a typical feature of breakdown. The reason is that in the 35% sample, the conduction path is blocked by a thin layer of silicone rubber matrix, as shown in Fig. 3(a,b) by yellow lines. This layer restricts the rise of current with the increase of applied electric field *E*. When *E* is high enough, fillers in the blocked conduction path present much lower resistivity due to their nonlinearity while the silicone layer remains a high volume resistivity. Under this circumstance, a great part of applied voltage is transfer to the thin silicone layer according to the voltage division rule. Hence the field strength on the layer is severely high and when it is beyond the critical breakdown intensity, the layer would be punctured. At this time in the conduction path, both the insulation layer and the fillers are of low resistivity thus the current increases sharply.

The V-I characteristics of 35% sample were repeatedly measured right after the first measurement which presents breakdown feature. All the subsequent measured V-I curves are the same, as Fig. 4 presents: the switching field is greatly reduced and the curve exhibits typical nonlinear feature. The simulated V-I curve is also identical to that. This measurement result can be explained as follows. In the subsequent measurement, the current is very likely to select the same path to flow through, which is verified by simulation (Supplementary Note 1, Fig. S1). Then in the conduction path the polymer layer once has been punctured is conductive, which shortens the path instead of blocking it. At this time the conduction path is relative straightforward as Fig. 3 shows. It is similar with the paths in the fully percolated case and develops without the hindering of insulation layer. Therefore the 35% sample presents a much lower switching field close to the 43% sample in the subsequent measurement.

Several days later the V-I characteristic of the same 35% sample is measured again, the result is the same as the first measurement with breakdown feature. This indicates that the previous breakdown is not destructive and the polymer layer can recover to original dielectric strength. The reason is that the silicone rubber matrix has relative well fluidity thus it can cure itself in few days if the breakdown is not severe in a small region. But when measured again, the polymer layer once has broken down is more susceptible despite of the recovery. Thus the same conduction path forms and the same result as the first measurement is observed. Similar self-healing phenomenon in silicone rubber has been observed by former research^27^.

The discussion above indicates that the local polymer layer breakdown has considerable effect on the nonlinear conducting behavior of the composites, especially when the composite is nearly percolated like the 35% sample.

### Further Lower Filler Concentration below Percolation

When the filler concentration is reduced to 30%vol, the conducting behavior of the composite varies. The sample presents much higher breakdown electric field beyond the maximum output of the source. The reason is that in the 30% sample, a conduction path is even harder to form. More and thicker insulation layers exist in a potential path as shown in Fig. 3(a,b) by the yellow lines. To puncture the layers, much higher applied field is required. Thus the sample failed to exhibit nonlinear conducting behavior and even the breakdown feature is not observed in the experiment. Then another source which is specific for the measurement of breakdown voltage was applied and the breakdown field of the sample was measured to be about 5 kV/mm.

Right after the breakdown experiment, the V-I characteristic of the same sample was repeatedly measured again but this time we got a nearly linear V-I curve (Supplementary Note 2, Fig. S2). This indicates that severe insulation damage has occurred in the sample and the breakdown region may be even larger than that in Fig. 3. As large regions of insulation layers became conductive after punctured, the subsequent measured V-I characteristics were more close to linearity. For the 25% sample, the breakdown field is even higher than 8 V/mm and a V-I characteristic with more obvious linearity was observed after breakdown measurement.

### Determination of the Percolation Threshold

From the experimental measured V-I curves, the switching fields (breakdown fields) of the samples are presented in Fig. 5 by the red line. We can see that the *E*_b_ of the composite exhibits nearly continuous change with the filler concentration from 47% to 35%. For the 35% sample, the conduction path has basically formed and the composite also possesses typical nonlinearity after puncturing of a thin polymer layer. Moreover, the breakdown won’t bring about great damage to the insulation and is resumable. Thus we can attribute the near percolated composites like the 35% sample to the percolated case. But when the filler concentration is reduced to 30%, the conducting behavior is quite different: not only the switching field is much higher but also the sample fails to exhibit nonlinear property after breakdown, because the insulation damage is severe.

It is clear that when the filler concentration goes from 30% to 35%, the property of the composite has a sharp change. Therefore, the percolation threshold is expected to be between 30% and 35%. To get a more specific value and analyze the whole percolation process, the paper has calculated the breakdown field *E*_b_ (switching field for percolated cases) of the composites with volume fraction from 5%vol to 55%vol through simulation, shown as the black line in Fig. 5. The simulation also demonstrates that sharp change occurs between the volume fraction of 30% and 35%. The critical point seems to be 33%vol, after which the breakdown field decreases continuously and the composites begin to present nonlinear behavior. Thus we can determine the percolation threshold of the ZnO/silicone rubber composites as 33%vol and this value is identical to the percolation threshold that determined through experiment for composite with rounded SiC filler^25^.

### Percolation Process Analysis

Breakdown paths of composites with a wide range of filler concentrations are obtained through simulation. Figures with typical features were selected to demonstrate the whole percolation process, shown as Fig. 6. For composites with low filler concentration like the 5% case, the breakdown path looks like an electrical tree mainly developing through the insulation. In this way, the filler is of minor influence and the breakdown field is relative high. When fillers are in higher concentrations like 15%vol, they begin to guide the breakdown path as their volume resistivity is lower in nonlinear state and the current is more likely to flow through them. From the case of 15%vol to 30%vol the breakdown paths in the composite are all roundabout, with many thick insulation layers between the fillers. But the layer gradually becomes less and thinner as the filler concentration increases. Thus the breakdown field continuously decreases though the conduction path does not form yet. When the filler volume fraction reaches the key point of 33%, the conduction path has basically formed and hence there is a sharp decrease of the breakdown field. After that the composites can present excellent nonlinear behavior directly (for percolated cases) or after the breakdown of a thin silicone rubber layer (for near percolated cases). From that on, the layers becomes tinier and tinier until the filler concentration reaches about 39% when the layer almost vanishes in a conduction path. But at this time the path is quite roundabout. Then from the case of 40% to 46% the paths become more and more straightforward. Definitely during this process, the switching field keeps going down. It is the concentration of about 46% that a straight conduction path can always form in the composites. Under this circumstance the switching field becomes independent of the filler volume fraction as it reaches the minimum value of about 440 V/mm. This is close to the value that the switching voltage of a single boundary (3 V) divided by average grain size of 7 μm.

It is worth mentioning that the percolation threshold of 33%vol determined in this paper is only for the case of random-distributed rounded fillers with the diameter range of 60–80 μm. If the shape, diameter or distribution pattern of the filler changes, the percolation behavior and especially the critical threshold would be different. In our previous study^9^, the experimental determined threshold is about 31%vol for ZnO/silicone rubber composite with the filler diameter of 125–150 μm. The reason for this lower threshold value may be that greater fillers are more likely to form conduction paths. Also it is well known that for composite with fillers of high aspect ratio, such as fiber-like fillers, the percolation threshold could be much lower^18^. Finally the distribution pattern of the fillers is significant to the percolation behavior as well. If the fillers are manually controlled to align throughout the composite, a conduction path can be established even in a 10%vol sample^22^; if the fillers are controlled to form a periodic lattice, the composite would not percolate at all. For composites with randomly distributed fillers, the analysis of percolation process involves the problem of probability. The percolation threshold of 33%vol doesn’t mean that in the 30% sample a conduction path will never occur while for the 35% sample a conduction path surely exists. Instead it indicates that in the 30% sample a conduction path is not likely to exist and in 35% sample the path is in great potential to form.

In general, the percolation behavior discussed above is in the sense of probability and is microstructure-specific, the analysis approach in this paper may be helpful to the research of nonlinear composites but the threshold behavior is not universal.

## Conclusion

The percolation characteristics of the nonlinear composites dielectrics are different from the traditional conducting composites. Aiming at this issue, a new model based on Voronoi network is proposed and agrees well to the measured nonlinear conducting behavior of ZnO-silicone rubber composites, especially for the near percolated case with local insulation puncturing phenomena. The percolation process of the composite is well explained through both experiment and simulation result. The percolation threshold of the composite is determined as 33%, which accords with the expectation of relative high threshold value for nonlinear composites.

The work in this paper also provides an effective method to simulate the polymer composites. In the model, the shape and size of the filler can be more consistent to the practical situation and the property of the filler, the insulation matrix as well as the contact interface can be together taken in to consideration. This is helpful in studying novel composite materials.

## Method

### Simulation

#### Basic Voronoi Network Model

The model is based on the Voronoi network, which can effectively simulate the electrical property of ZnO varistors on the basis of interior grain connection^28^. In ZnO varistor simulation, shown as Fig. 7(a), each polygon lattice represents a conductive ZnO grain whose resistance can be attributed to the grain boundary. The shared edges between two polygons are the grain boundaries which can be seen as nonlinear impedance and mainly determine the electrical property of the varistor. Thus the simulation of electrical property of ZnO varistor can be transformed into solving a nonlinear impedance network circuit, shown as Fig. 7(b), in which edges of the polygons represent nonlinear impedances and centers of the polygons are nodes of the circuit.

**Figure 1:**
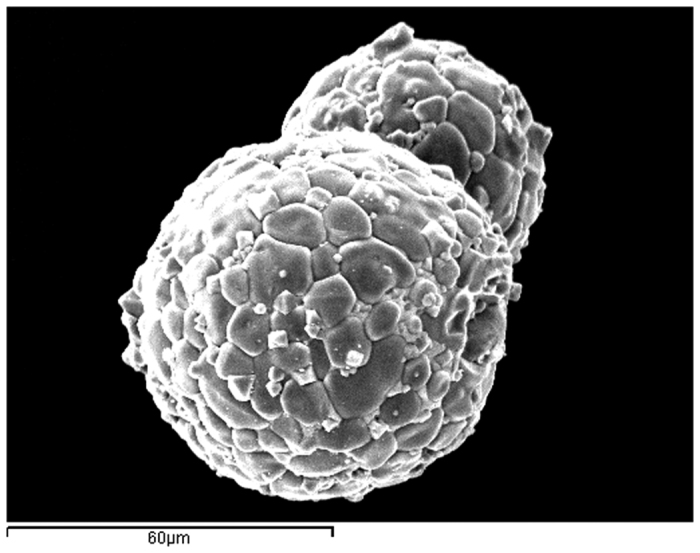
SEM image of two contacted ZnO microspherical varistors.

**Figure 2:**
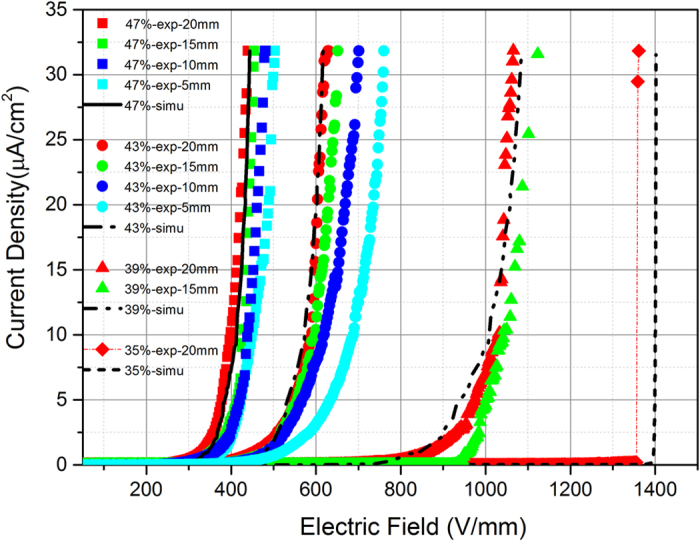
Nonlinear V-I characteristics of ZnO-microvaristor samples with filler concentrations of 35%, 39%, 43%, 47%vol in simulation (lines) and experiment (scattered points) under different areas of electrodes.

**Figure 3:**
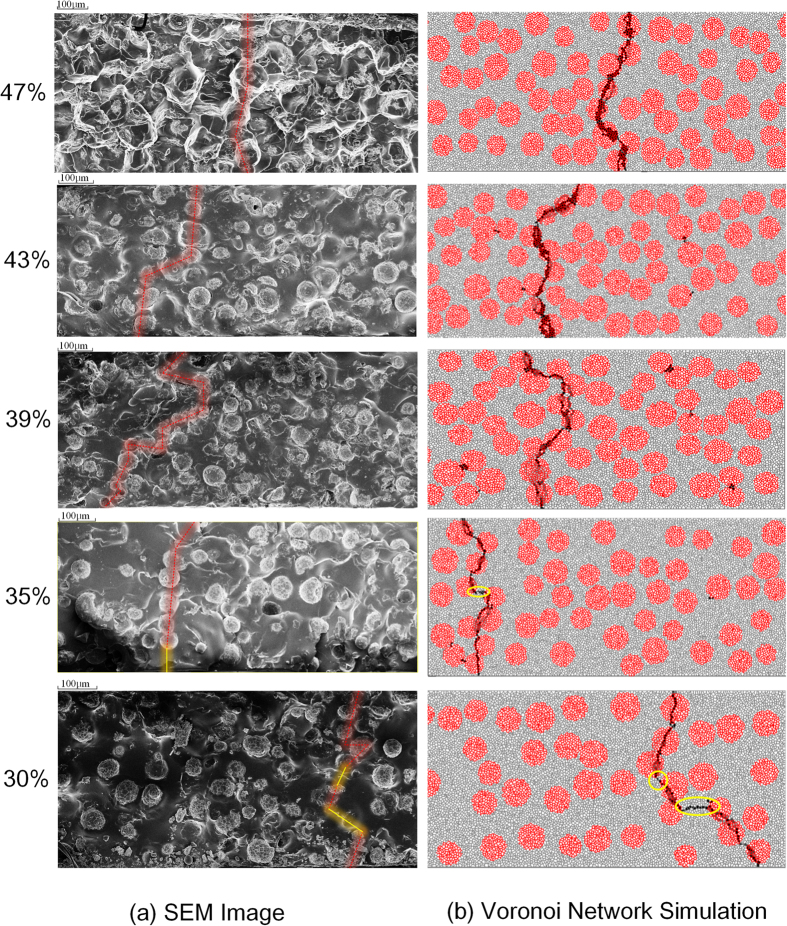
(**a**) SEM images and possible conduction path (**b**) Voronoi network Simulation of filler and current density distribution in ZnO-microvaristor composites.

**Figure 4:**
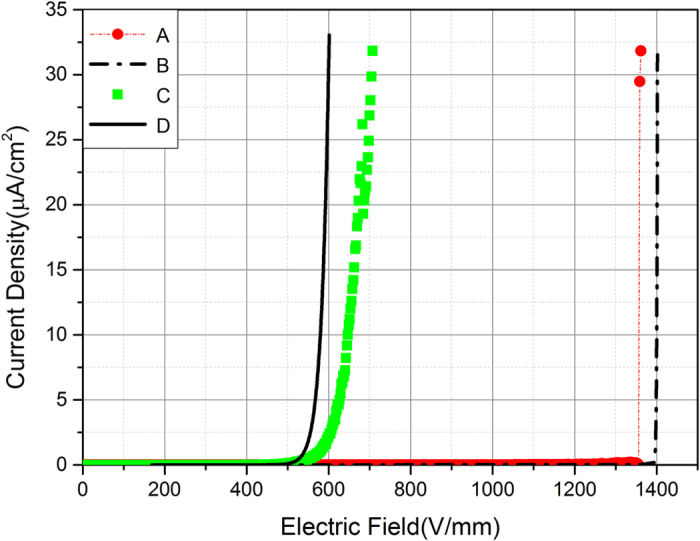
*J*(*E*) characteristics of the same 35%vol ZnO composite sample in the first measurement by experiment (Curve **A**) and simulation (Curve **B**), and the subsequent measurement by experiment (Curve **C**) and simulation (Curve **D**).

**Figure 5:**
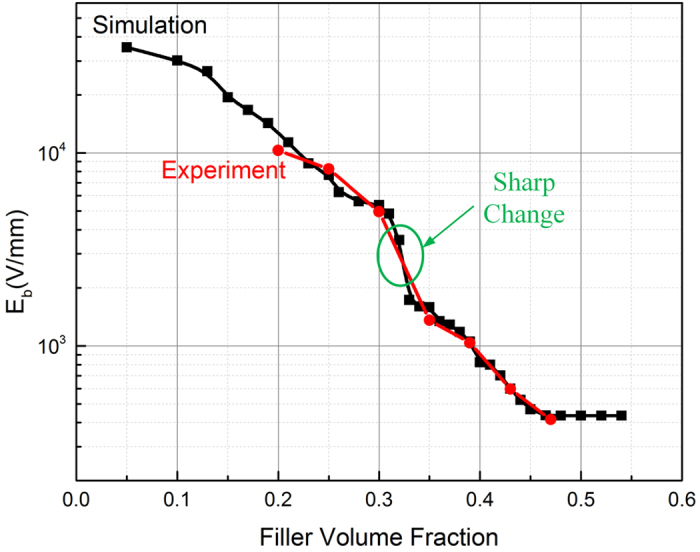
Simulated breakdown field of ZnO-silicone rubber composites with filler volume fraction from 5% to 55% (black curve simulation curve and red curve for experiment result).

**Figure 6:**
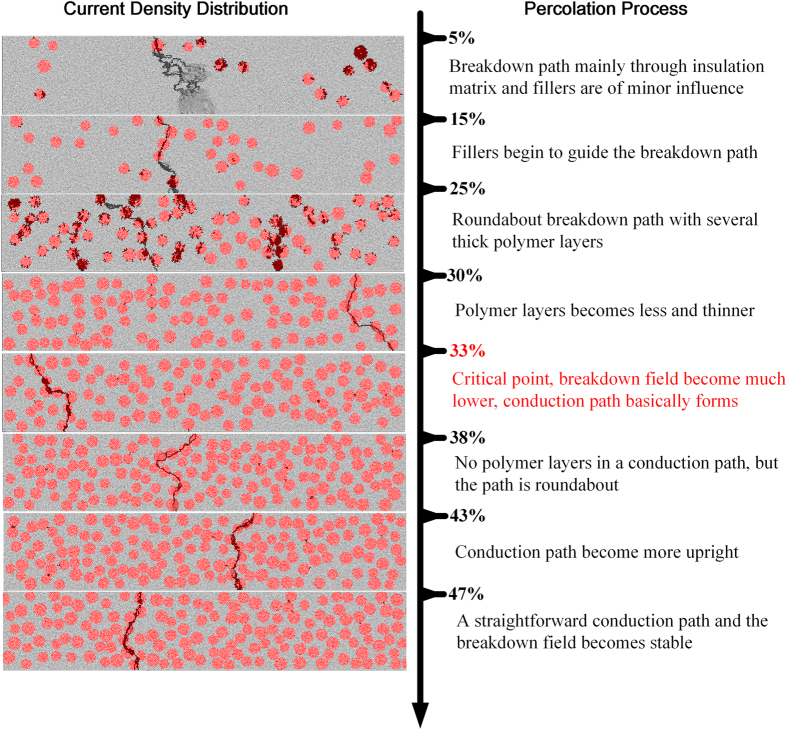
Schematic diagram of the percolation process based on the filler and current density distribution obtained from simulation.

**Figure 7:**
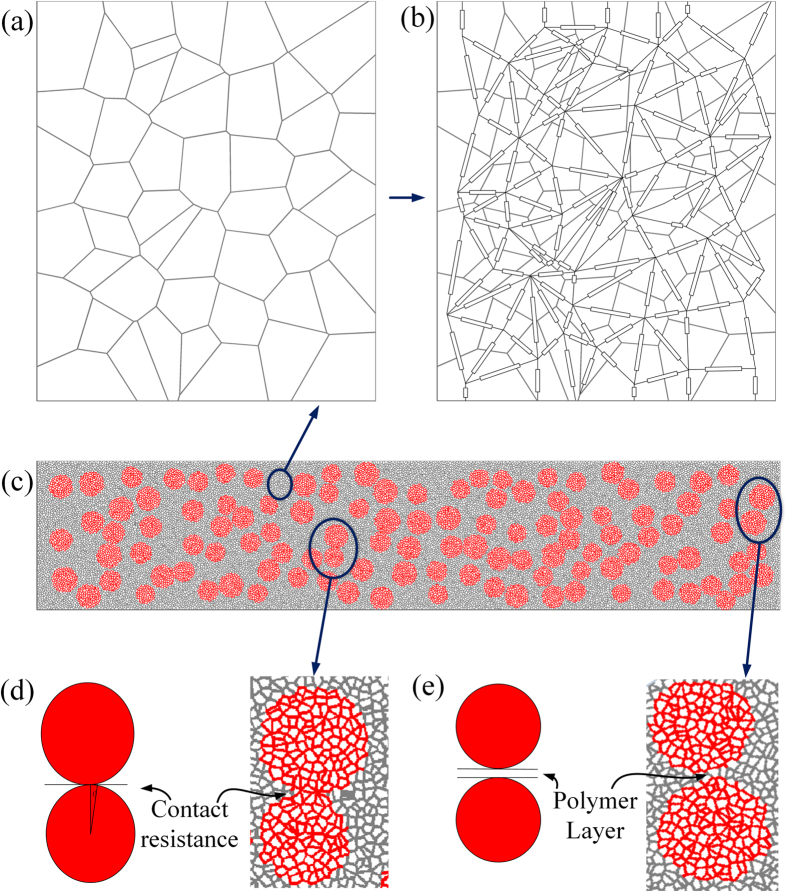
Schematic diagram of the simulation based on Voronoi Network (**a**) Geometry structure of the Voronoi network; (**b**) Circuit model developed based on the network (**c**) Simulation for filler distribution in composite; (**d**) A close contact between two rounded fillers (**e**) A thin polymer layer between two adjacent fillers.

The same method can be used to simulate the ZnO microvaristor-insulation matrix composites, shown as Fig. 7(c). The whole rectangular Voronoi region was firstly set up to represent the studied composites and then red circles randomly distributed in the region were the nonlinear fillers. Thus polygons in red lines inside the circles are grains in microvaristors. This is quite similar to the actual SEM image of a microspherical ZnO varistor shown as Fig. 1. Polygons in black lines outside the circles are insulation matrix. The matrix is deliberately partitioned into polygon elements with the same size as ZnO grains for the simplicity of calculation.

#### Models for ZnO Microvaristor and Insulation Matrix

Once the interior geometry feature of the nonlinear composite has been settled, the electrical parameters of the impedance network can be set according to the material property. Edges that present the insulation matrix are set as normal permittivity and a conductivity with breakdown characteristics as Equation (1) demonstrates.


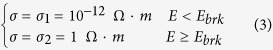


where *E* is the electric field that local insulation matrix bears and *E*_brk_ is the puncture field intensity of the material.

Edges that represent ZnO grains are set with a fixed capacitance (field dependent permittivity of ZnO fillers are ignored here) and a typical nonlinear V-I characteristic with the switching voltage of about 3 V, which is derived from experimentally measured V-I curves of single microvaristor fillers (Supplementary Note 3). In addition to the nonlinear part, the resistance of the grain, the thick Bi_2_O_3_-rich intergranular layer and the directly contacting point are also considered in the model according to the three types of grain boundary structure^29^. They can be processed together with the nonlinear conducting property of the boundaries as a whole dynamic resistance in the Voronoi impedance network.

#### Models for Interfaces between the Fillers

For contact interfaces between rounded fillers, there is only one type of interface resulting from a tangent contact shown as Fig. 7(d). This interface is especially significant for nonlinear fillers whose nonlinearity originates from particle-particle contacts such as SiC, C, Al^30^. But for ZnO microvaristors whose nonlinearity mainly comes from intrinsic grain boundary, we ignore the nonlinearity of the interface and only consider the contact resistance *R*_c_. *R*_c_ can be calculated together with the volume resistivity *ρ* of the particle as the apparent resistivity *ρ*_*a*_^31^, as shown in Equation (4):


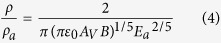


where *E*_*a*_ is the average electric field applied to the composite, *A*_*V*_is a constant that can be set as 1.28 for hard fillers with small contact angles, *B* is an coefficient relevant to the filler’s Young’s modulus *Y* and Poisson’s ratio *υ* as 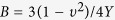.

When fillers are not in close contact with each other, there are thin polymer layers between the particles shown as Fig. 7(e). For traditional conducting composite with near-percolated filler distribution, conduction is dominated by electron tunneling across the thin polymer layers separating the conducting particles^32^,^33^. However, for nonlinear composites, the conductivities of the fillers are much lower even in nonlinear state because they have graded the electric field and bear relative low field intensity. Hence the tunneling conduction between nonlinear fillers is far weaker than that between conducting fillers. Hence the tunneling effect is not taken into consideration in this model. However, both in tradition conducting composites and nonlinear composites the polymer layers bear very high electric field in near percolated case and the field may be high enough to puncture the polymer layers. Thus the conductivity of the insulation matrix is set as Equation (3) to take this case into consideration.

#### Simulation Conditions

The simulation conditions are set to be consistent with the practical experiment. The average size of the Voronoi polygons is set as the average grain size of the microvaristor and the width of the simulation region is set as the thickness of the practical composite sample. For better comparison, the length of the simulation region is set close to the minimum diameter of the golden electrodes which are deposited on the surfaces of the composited sample. To rule out the contingency due to the random distribution of the fillers, repeated calculation for a same case is necessary. Specifically, we repeatedly generate new filler distribution and simulate its V-I characteristic under the same condition. After that a group of V-I curves are acquired and the one with minimum switching voltage is selected as the final result because the current will always flow through the shortest path. In this paper, all the simulation obtained V-I curves are from the result of 10 times of calculation under the same condition.

The experimental voltage source can provide step voltage to 1100 V and the compliance current can be set to 0.1 mA, corresponding to maximum current density of about 30 μA/cm^2^ on the sample. For the simplicity of the simulation, a current source with step current increase to 30 μA/cm^2^ is adopted to calculate the V-I characteristics of the composites. Thus the experimental and simulation nonlinear J-E curve can be directly compared.

### Experimental

#### Preparation of ZnO fillers and ZnO-silicone rubber composites

ZnO microspherical varistors samples were prepared based on conventional formula and preparation procedures of ZnO varistors^34^,^35^,^36^ as 95 mol% ZnO + 1.0 mol% Bi_2_O_3_ + 0.5 mol% MnO_2_ + 1.0 mol% Co_2_O_3_  + 0.4 mol% Cr_2_O_3_ + 1 mol% Sb_2_O_3_ + 1.0 mol% SiO_2_ and 0.1 mol% Al_2_O_3_. The mixed powder was sent into ball mill and blended in anhydrous alcohol for 8 hours. After addition of organic binder, the aqueous slurry was processed by the spray-dried machine and the mixture was granulated into spherical particles. Hereafter, the microspheres were sintered at the temperature of 1200 °C for 4.5 h with the heating rate of 0.55 ^o^C/min and cooling rate of 1.6 ^o^C/min. After disagglomeration and further sifted, ZnO microvaristor filler with the diameter range of 60–80 μm were acquired.

ZnO microvaristor composites based on silicone matrix were then prepared. The silicone and vulcanizing agent were mixed in 0.8 wt% with tetrahydrofuran solvent and blended by a high torque blender for about 20 minutes. After silicone was fully dissolved in the solvent, ZnO microvaristor powders were poured into the liquor and the blending continued for 40 minutes. Then the mixture was dried in a vacuum oven for more than 10 hours till the solvents fully volatilized. After that was the process of vulcanization. Each time 3 g mixture was pressed by the vulcanizing machine in the pressure of 15 MPa at 170 °C for 15 minutes and then naturally cooled to room temperature in the same pressure. The acquired silicone rubber composite sample is about 0.5 mm in thickness and 20 mm in diameter.

#### Sample Grouping and Measurement

ZnO microvaristor/silicone rubber composite samples with six different filler concentration were prepared. ZnO fillers were mixed into silicone matrix in the volume fractions of 20%, 25%, 30%, 35%, 39%, 43%, 47%. Golden electrodes with diameters of 5 mm, 10 mm, 15 mm, 20 mm were successively deposited on to the surfaces of the sample. *I*(*U*) measurement for the sample was simply performed by power source (Kiethley 2410C) right after each electrode is deposited. The parameter of the voltage source has been introduced in the section of Simulation Condition. Thus for each composite sample we have acquired four *I*(*U*) curves under different electrode areas. The *J*(*E*) characteristic of the samples are acquired from the measured *I*(*U*) curve by Equations (5).


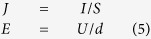


where *d* and *S* are the thickness and cross-section of the sample^37^.

## Additional Information

**How to cite this article**: Yang, X. *et al.* Understanding the Percolation Characteristics of Nonlinear Composite Dielectrics. *Sci. Rep.* **6**, 30597; doi: 10.1038/srep30597 (2016).

## Supplementary Material

Supplementary Information
