# Supplementary material for: Understanding the Percolation Characteristics of Nonlinear Composite Dielectrics
Source: Sci Rep. 2016 Aug 1;6:30597. doi: 10.1038/srep30597 (PMC4967925; doi:10.1038/srep30597)
Supplement: Supplementary Information [file srep30597-s1.pdf]

Supplementary Information

Understanding the Percolation Characteristics of Nonlinear Composite Dielectrics

Xiao Yang, Jun Hu,and Jinliang He

State Key Lab of Power Systems, Department of Electrical Engineering, Tsinghua University, Beijing, 100084, China

Correspondence and requests for materials should be addressed to J. H. (hjun@tsinghua.edu.cn) or J.L.H. (email: hejl@tsinghua.edu.cn.)

Supplementary Note 1: Simulation of current density distribution diagram of 35%vol composites

The simulation diagram of filler and current distribution of 35%vol sample in the first and subsequent measurement are shown as Figure Fig.S1. We can see that the two subfigures are in little difference and this indicate that the conduction path after the first measurement remains almost the same.

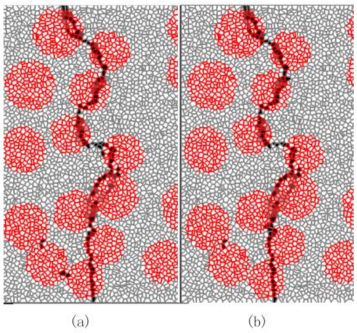

Supplementary Fig. S1. Current density distribution diagram of 35%vol composites in (a) first and (b) subsequent simulation.

Supplementary Note 2: J-E characteristics of 30%vol sample after breakdown

J-E characteristic of the 30% sample after the breakdown test is shown as Fig.S2. Compared to the typical nonlinear conducting property of ZnO micromaristor

composites, this acquired J-E curve presents almost linear property, which means that severe insulation damage might occur in the breakdown test.

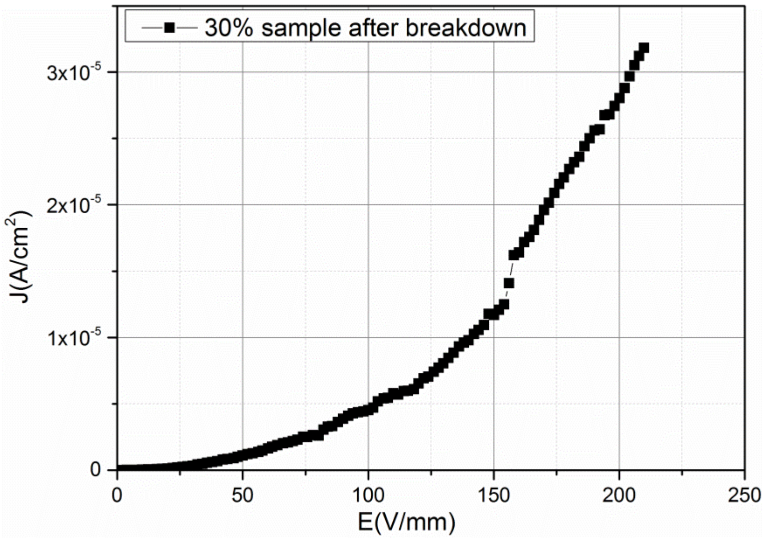

Supplementary Fig. S2. Measured J-E characteristics of the 30% sample after breakdown.

Supplementary Note 3: Measurement of I-V characteristics of single ZnO microvaristor fillers and the determination of the I-V curve of the grain boundary.

Microprobe platform (Cascade Microtech Summit 11000B-M) was applied to measure the I-V characteristics of single ZnO microvaristor fillers, shown as Figure Fig.S3. Particles can be observed through microscope and the microprobe with gliding tip radius of about 0.2 μm can directly operate on the particles. The microprobes can be connected with power source for measurement. Fillers are dispersed on the copper foil and the foil is grounded. A probe tip functioned as a positive electrode is tightly pointed against the top of the filler, which guarantees a good electrical contact between the fillers and the both electrodes. In this way I-V characteristics of single ZnO microvaristor fillers can be easily acquired.

However in the simulation, we use the I-V characteristic of a single grain boundary instead of microvaristor filler. Thus an averaged I-V curve of the grain

boundary is expected to be determined through the curves of the fillers. First we simply define average electric field E applied on a single filler by Equation (S1).

$$E = U / d \tag{S1}$$

where d is the diameter of the filler. Thus $I(E)$ characteristics of the fillers are acquired. The measured $I(E)$ curves of a number of fillers are in little discrepancy and are averaged shown as Fig.S4(a). Then from the SEM images of the fillers we can get an approximate averaged grain size of the fillers as about 6μm. It is well known that for ZnO microvaristors the grain itself is of little resistivity and the applied voltage is mainly on the grain boundary. Thus the averaged electric field applied on the filler multiplied by the averaged grain size can be considered as the voltage applied on each grain boundary in a filler. Therefore we can get an averaged I-V characteristic of the grain boundary, shown in Fig.S4(b). From Fig.S4(b) we can see that the switching field of the averaged curve is about 3V, which is a typical value for the grain boundary determined by former studies[1,2,3].

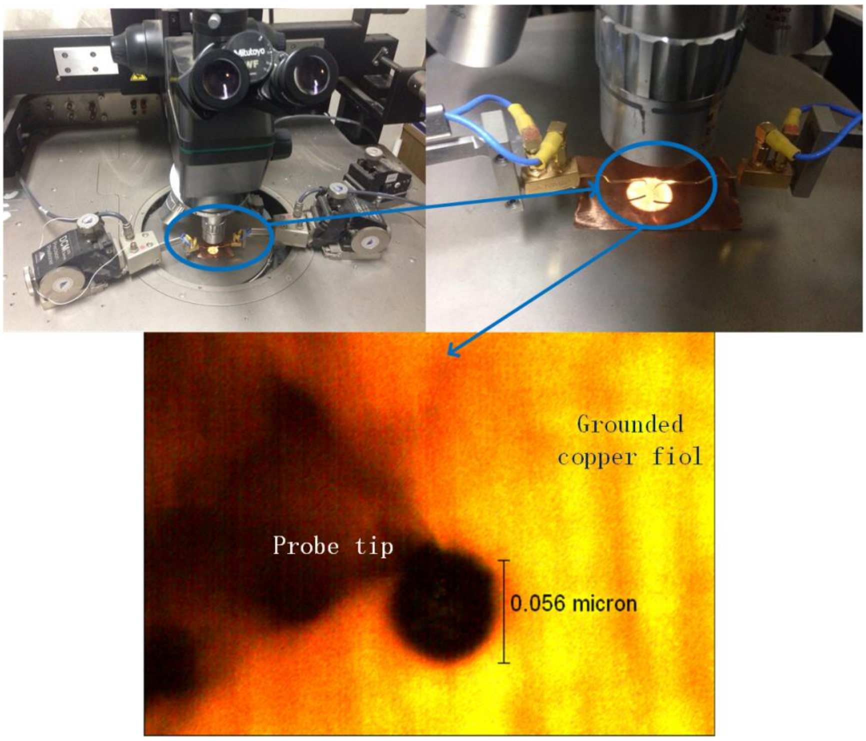

Supplementary Fig. S3. Measurement of V-I characteristics of single ZnO microvarisotr fillers through a microprobe platform.

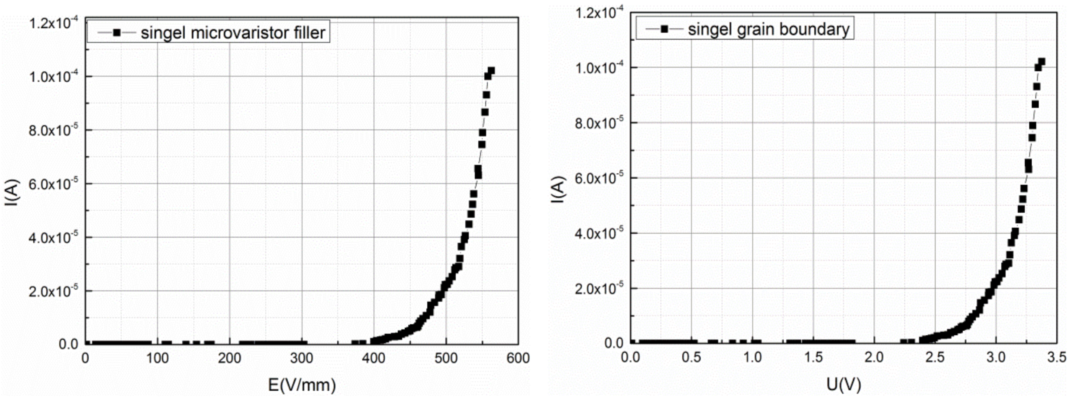

(a) **(b)**

Supplementary Fig. S4. (a) Averaged I-E characteristics of a single microvaristor filler. (b) Averaged I-V characteristics of a single grain boundary.

Supplementary References

1. Levinson L M, Philipp H R. Zinc Oxide Varistors—A Review. *Am. Ceram. Soc. Bull.*, 1986, 65(4):639-646.
2. Levinson L M. Advances in Varistor Technology. *Am. Ceram. Soc. Bull., 1989*, 68(4):866-868.
3. Einzinger R. Metal Oxide Varistors. *Ann. Rev. Mater. Sci.*, 1987, 17:299-321.
